# Nutraceutical Curcumin with Promising Protection against Herpesvirus Infections and Their Associated Inflammation: Mechanisms and Pathways

**DOI:** 10.3390/microorganisms9020292

**Published:** 2021-01-31

**Authors:** Miroslava Šudomová, Sherif T. S. Hassan

**Affiliations:** 1 Museum of Literature in Moravia, Klášter 1, 66461 Rajhrad, Czech Republic; sudomova@post.cz; 2 Department of Applied Ecology, Faculty of Environmental Sciences, Czech University of Life Sciences Prague, Kamýcká 129, 6-Suchdol, 16500 Prague, Czech Republic

**Keywords:** curcumin, herpesviruses, phenolics, inflammation, mechanisms and pathways, viral infections, *Curcuma longa* L.

## Abstract

Herpesviruses are DNA viruses that infect humans and animals with the ability to induce latent and lytic infections in their hosts, causing critical health complications. The enrolment of nutraceutical anti-herpesvirus drugs in clinical investigations with promising levels of reduced resistance, free or minimal cellular toxicity, and diverse mechanisms of action might be an effective way to defeat challenges that hurdle the progress of anti-herpesvirus drug development, including the problems with drug resistance and recurrent infections. Therefore, in this review, we aim to hunt down all investigations that feature the curative properties of curcumin, a principal bioactive phenolic compound of the spice turmeric, in regard to various human and animal herpesvirus infections and inflammation connected with these diseases. Curcumin was explored with potent antiherpetic actions against herpes simplex virus type 1 and type 2, human cytomegalovirus, Kaposi’s sarcoma-associated herpesvirus, Epstein–Barr virus, bovine herpesvirus 1, and pseudorabies virus. The mechanisms and pathways by which curcumin inhibits anti-herpesvirus activities by targeting multiple steps in herpesvirus life/infectious cycle are emphasized. Improved strategies to overcome bioavailability challenges that limit its use in clinical practice, along with approaches and new directions to enhance the anti-herpesvirus efficacy of this compound, are also reviewed. According to the reviewed studies, this paper presents curcumin as a promising natural drug for the prevention and treatment of herpesvirus infections and their associated inflammatory diseases.

## 1. Introduction

**Figure 1:**
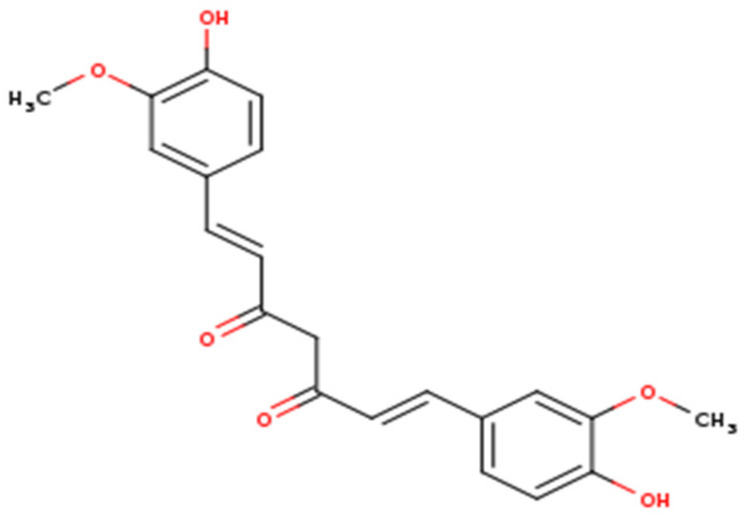
Chemical structure of curcumin.

Infection with herpesviruses is frequently observed in humans and animals, inducing a range of diseases from moderate uncomplicated mucocutaneous infection to those that are life-threatening [1,2]. Herpesviruses belong to the family of *Herpesviridae*, which is a large family of DNA viruses with severely contagious properties that use a strategy of infection known as travel and hide [3,4]. These viruses share the feature of forming lifelong infections in a latent phase with the potential of periodic reactivation. During the life cycle, herpesviruses typically infect different cell types in various tissues, and therefore the sub-classification of these pathogens is partially based on their cell and tissue tropism [5,6,7]. So far, the herpesvirus family is divided into three subfamilies, the Alpha-, Beta-, and *Gammaherpesvirinae*, which include diverse types of herpesvirus known to infect humans. The morbidity and mortality of these viruses are mainly linked with the immunocompromised hosts. However, herpes simplex virus (HSV), varicella-zoster virus (VZV), and Epstein–Barr virus (EBV) were reported with a significant disease burden in people with normal immune function [8,9]. For decades, integrated management of herpesvirus infections remains the main challenge in virology research, where these infections are still incurable due to the viral latency, the problem of recurrent infections, and the resistance to antiherpetic drugs [10,11,12]. Antiviral natural products have gained immense consideration in herpesvirus research after the Nobel Prize in Physiology or Medicine (in 2015) was awarded for the discovery of the natural anti-malarial drug artemisinin. Thus, natural anti-infective agents might pave the road for defeating the problem of drug resistance as well as provide effective and safe treatment of infections caused by herpesviruses [13,14,15,16]. Curcumin, a natural phenolic compound found in the *Curcuma longa* L. (*Zingiberaceae*) (turmeric), could help eliminate certain viruses by various mechanisms of action (Figure 1). Therefore, in this review, we aim to collect all in vitro and in vivo studies of curcumin that show its promising inhibitory properties against various types of herpesvirus infections and inflammation linked with these diseases. Moreover, the mechanisms and pathways by which this compound causes anti-herpesvirus effects are discussed. Challenges with its bioavailability and strategies to improve its anti-herpesvirus properties are also reviewed. 

Databases such as Web of Science, PubMed, Scopus, SciFinder, Google Scholar, and ScienceDirect were used to perform the literature search, using terms that define curcumin, herpesvirus infections (human and animal herpesviruses), and their associated inflammation. The literature search has covered studies (in vitro and in vivo investigations) published in the years from 1999 to 2020.

## 2. Curcumin as an Antiviral Agent with Proven Health Benefits

Curcumin is the key component of the yellow pigment and the main bioactive molecule of turmeric [17]. Chemically, this compound belongs to the class of natural phenolic compounds and has been broadly identified in diverse *Curcuma* spp. In 1910, curcumin was characterized as a symmetrical molecule of two 4-hydroxy-3-methoxyphenyl rings fastened by α,β-unsaturated carbonyl groups [18,19], while its synthesis was defined in 1913 [20]. Curcumin has been employed widely in the traditional medicine systems of various countries and regions in the world [21,22]. Since the complete information about chemical structure and synthesis is acquired, curcumin has been extensively studied in various biological assays and has proven to induce numerous pharmacological and beneficial impacts on human health, including but not limited to the potential treatment of various viral infections such as human immunodeficiency virus, hepatitis B virus, hepatitis C virus, influenza A virus, human papillomavirus, respiratory syncytial virus, arboviruses, and noroviruses [23,24,25,26,27]. Unlike the notable antimicrobial actions, this biomolecule induces several biological effects including but not limited to antioxidant, anti-inflammatory, and anticancer properties [28,29].

## 3. Anti-Herpesvirus Drugs Used in Clinics

To date, acyclovir and other antiviral nucleoside analogs are used in the first-line treatment strategy for herpesvirus infections. The potent antiherpetic properties of these drugs are related to their ability to block viral replication [30,31]. However, these drugs do not heal the disease while reducing the duration of symptoms and accelerating the recovery of lesions and epithelial damage leading to a return to normal condition. The extensive use of these drugs in the therapy has formed the problem of drug resistance, leading to noticeable treatment failure [32,33,34]. While various ongoing studies have attempted to produce herpesvirus vaccination, no vaccines or antiviral drugs have been approved for the prevention of herpesvirus infections so far [35,36]. Therefore, there is an urgent demand for developing novel antiherpetic drugs that could effectively cure or prevent herpesvirus infections with less resistance, diminished adverse effects, low toxicity, and improved stability [37,38].

## 4. Role of Curcumin in Inhibition of Herpes Simplex Virus Infections

It is known that nonclinical virology studies can assist in dose selection and study design to offer verification of concept and data validating an antiviral claim [39,40]. Consequently, in this section, we aim to embody all available data obtained from nonclinical investigations (in vitro and in vivo studies) that highlight the antiviral properties of curcumin, curcumin metal complexes, and curcumin formulations against the pathogenic herpes simplex virus (HSV) with an emphasis on the effective concentrations or doses and mechanisms of action (Table 1). The alpha-herpesvirus HSV is one of the most prevalent infections in humans, and therefore studies on this virus are intensively performed [41,42]. HSV is classified into two types, HSV-1 and HSV-2. HSV-1 is widely transmitted by oral-to-oral contact to induce oral herpes and in some cases, can also generate genital herpes. Genital herpes is a sexually transmitted disease triggered by HSV-2 [43,44]. Moreover, infection with HSV-2 enhances the risk of transmitting infection with the human immunodeficiency virus (HIV). Most oral and genital herpes infections are asymptomatic and persist lifelong in the host [45,46]. Considering the antiviral capacity of curcumin, defeating the viral infections is one of the main bio-functions reported for this compound via targeting of the viral entry and viral components that are crucial for viral replication as well as other cellular or molecular processes that are involved in the viral life/infectious cycle [47,48,49]. 

**Figure 2:**
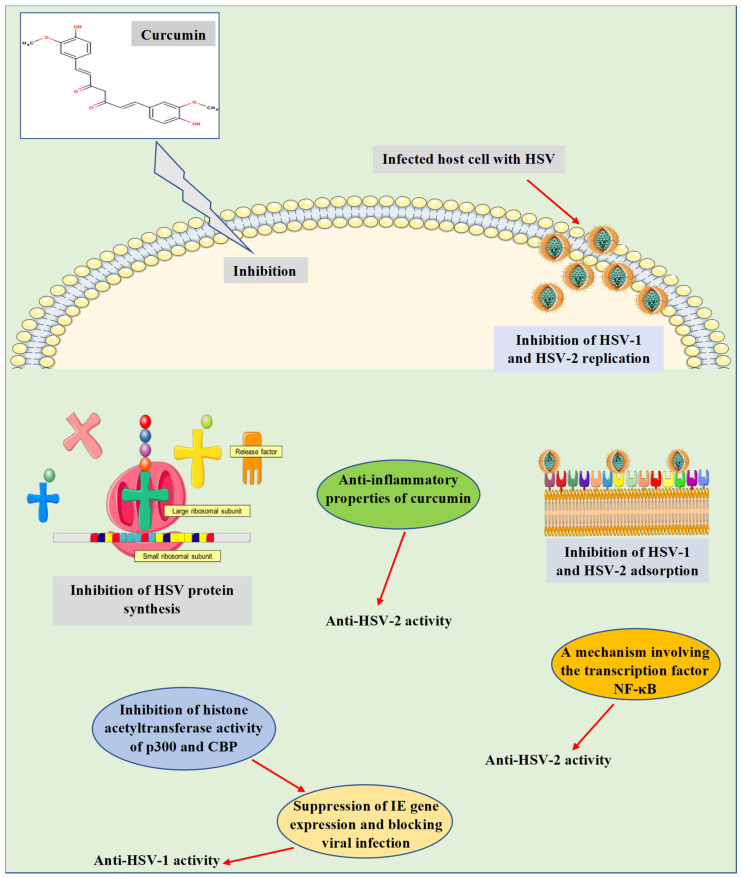
Antiviral properties and mechanisms of action of curcumin against herpes simplex virus infections. The presented mechanisms have been explored by in vitro and in vivo studies. CBP, CREB-binding protein; HSV-1, herpes simplex virus 1; HSV-2, herpes simplex virus 2; IE gene, immediate early gene; NF-κB, nuclear factor kappa B.

Consequently, the mechanisms by which curcumin induces anti-HSV actions were investigated in depth in various in vitro and in vivo studies (Figure 2). These mechanisms were found to relate to the ability of curcumin to hinder a range of cellular and molecular processes, which are essential for viral gene expression, replication, and pathogenesis. For instance, curcumin was detected to inhibit IE gene expression and hindering HSV-1 infection by employing a mechanism independent of the transcriptional coactivator proteins p300/CBP histone acetyltransferase activity to impact the viral transactivator protein VP16-mediated enlistment of RNA polymerase II to IE gene promoters [50]. In preclinical studies (in vitro and in vivo), Bourne et al. [51] stated that curcumin has effectively inhibited the replication of HSV-2. Besides, curcumin was reported to inhibit the replication of HSV-2 through a mechanism involving the transcription factor NF-κB [52]. Additional mechanisms were also verified in an in vitro experiment via inhibiting HSV-1 and HSV-2 adsorption [53]. In an in vivo investigation, the anti-inflammatory properties of curcumin nanoparticles were claimed to be one of the mechanisms responsible for inhibiting the infectivity and replication of HSV-2 [54]. Curcumin metal complexes such as gallium-curcumin and copper-curcumin were studied by Zandi et al. [55], where both complexes were identified to inhibit the replication of HSV-1. However, the mechanism of action has not been revealed, and further investigation needs to be performed. Recently, Treml and colleagues [13] have stated that the hydroxyl groups and phenyl rings of phenolic compounds, including curcumin, are responsible for the induced anti-HSV properties reported for these molecules. This has been probed in various structure-activity relationship analyses.

## 5. Curcumin Targets Thymidine Kinase Encoded by Herpes Simplex Virus

HSV encodes a set of enzymes that are essential for viral replication along with numerous enzymes involved in nucleotide metabolism that are not necessary for viral replication [2,13]. Such enzymes have been employed in various therapeutic strategies as valuable drug targets useful for the therapy of HSV diseases [2,40,56]. For instance, thymidine kinase (TK) is an essential enzyme that catalyzes the transfer of the gamma-phospho group of adenosine triphosphate (ATP) to thymidine, leading to generate thymidine monophosphate (dTMP). Thus, TK is a critical enzyme in the salvage pathway of pyrimidine synthesis [57]. This enzyme has been detected in both human and HSV with crucial molecular functions [58]. It has been well recognized that HSV-TK, which plays an imperative function in HSV pathogenesis, has a broad substrate specificity including pyrimidines and pyrimidine analogs (deoxycytidine, thymidine, and zidovudine) as well as purine (guanosine) analogs (acyclovir, ganciclovir, penciclovir, and buciclovir) [59,60]. Antiviral drugs that inhibit HSV DNA synthesis (for instance, acyclovir) require HSV-TK to be phosphorylated to monophosphate form. Cellular kinase enzymes then further phosphorylate the drug to the triphosphate form (an active form of the drug). Therefore, HSV TK plays an important role in the design of antiherpetic drugs as a critical mediator protein [61]. 

Recently, El-Halim et al. [62] have reported in an in silico assay the ability of curcumin to successfully bind to the active site of HSV-1 TK via forming hydrogen bonding and hydrophobic interactions with the amino acid residues of the enzyme active site. The results proposed hydroxyl and carbonyl groups along with phenyl rings of curcumin as functional groups that are accountable for the anti-HSV-1 TK activity. Indeed, the obtained results revealed the mechanism underlying the anti-HSV-1 activity of curcumin through the inhibition of HSV-1 TK. On the other hand, this study might pave the way towards additional investigations, where curcumin could act synergistically with acyclovir and with other nucleoside analogs, leading to improving the treatment efficacy of HSV infections. Unlike the crucial role of TK in HSV pathogenesis, HSV-TK in combination with ganciclovir has been detected to be a promising therapy for melanoma. This has been revealed in an in vivo study using the xenografted melanoma model, where curcumin combined with HSV-TK/ganciclovir efficiently impeded xenografted melanoma growth, which in turn, provided a potential therapeutic approach for improving gene therapy efficacy against skin cancer [63].

## 6. Role of Curcumin in Inhibition of Various Herpesviruses Infections

In addition to the remarkable antiviral properties against HSV infections, curcumin has been shown to inhibit other human and animal herpesviruses investigated in various assay systems. This section highlights all studies that have reported the antiviral properties of curcumin and curcumin formulations against human cytomegalovirus (HCMV), Kaposi’s sarcoma-associated herpesvirus (KSHV), Epstein–Barr virus (EBV), bovine herpesvirus 1 (BoHV-1), and pseudorabies virus (PRV) infections with a focus on concentrations or doses used along with the mechanisms of action (Table 2). 

**Figure 3:**
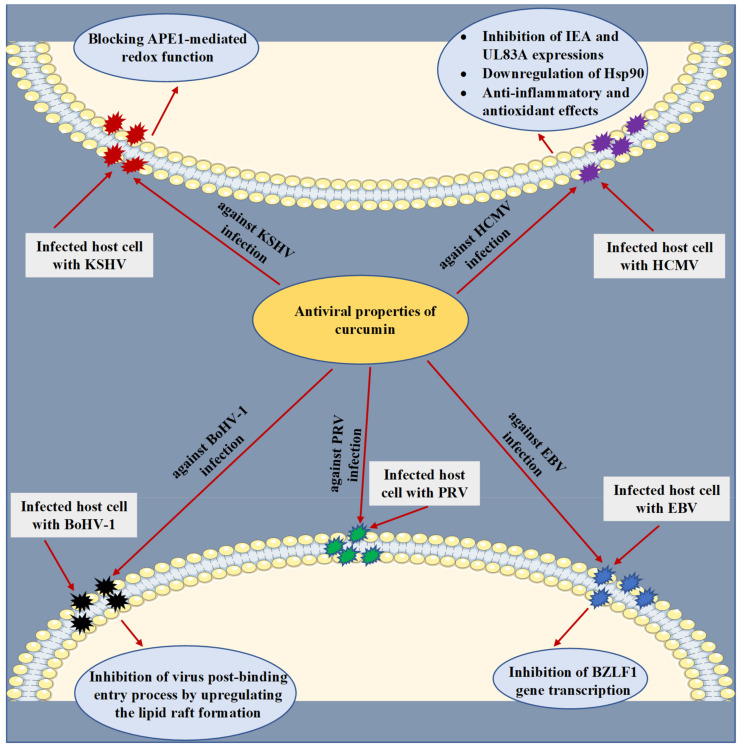
Antiviral properties and mechanisms of action of curcumin against human cytomegalovirus (HCMV), Kaposi’s sarcoma-associated herpesvirus (KSHV), Epstein–Barr virus (EBV), bovine herpesvirus 1 (BoHV-1), and pseudorabies virus (PRV) infections. The displayed mechanisms have been investigated by in vitro, in vivo, and in silico studies. APE1, apurinic/apyrimidinic endonuclease 1; Hsp90, heat shock protein 90; IEA, immediate early antigen.

HCMV is a human beta-herpesvirus that causes lifelong infection in the host and has been involved in the development of several diseases in humans, generating severe complications in immunocompromised individuals [64,65]. At various micromolar concentrations, the anti-HCMV activity of curcumin was explored in vitro, in an animal model (Balb/c mice) and in silico experiments. The underlying mechanisms were clarified through the capacity of curcumin to impede HCMV immediate early antigen (IEA) and UL83A expressions [66] and downregulate the heat shock protein 90 (Hsp90) [67] (Figure 3). Through a molecular docking analysis, curcumin was also found to accurately bind to the binding pocket of Hsp90 by establishing crucial contacts such as hydrogen bonds and hydrophobic interactions [67]. Moreover, the anti-inflammatory and antioxidant effects of curcumin were identified as possible mechanisms behind the antiviral action against HCMV [68]. 

KSHV is a gamma-herpesvirus (human herpesvirus 8) known to be one of the DNA tumor viruses involved in the etiology of human cancers [69,70]. It has been reported that the redox function of apurinic/apyrimidinic endonuclease 1 (APE1) plays a crucial role in the replication of KSHV [71,72]. Accordingly, Li et al. [73] examined the antiviral action of curcumin against the replication of KSHV. To reactivate the virus, primary effusive lymphoma (PEL)-BCBL-1 cells were latently infected with the virus and further were treated with 12-*O*-Tetradecanoyl-phorbol-13-acetate (TPA) and curcumin. The results indicated that treatment with curcumin at a concentration of 30 µM has led to significant blocking of KSHV reactivation by reducing expression of the switch gene replication and transcription activator (RTA) and the delayed-early gene K8. Additionally, with an IC_50_ value of 8.76 µM and an EC_50_ value of 6.68 µM, curcumin has notably reduced intracellular and extracellular KSHV genomic DNA levels, respectively. Eventually, the outcome of the study indicated that curcumin diminished the replication of KSHV by blocking APE1-mediated redox function. 

EBV is a gamma-herpesvirus known to infect humans that induces mononucleosis and is critically linked with multiple malignancies, including nasopharyngeal carcinoma, Burkitt lymphoma, Hodgkin lymphoma, gastric carcinoma, and posttransplant lymphoproliferative illnesses. Moreover, EBV was detected to be associated with some autoimmune diseases such as systemic sclerosis, multiple sclerosis, and systemic lupus erythematosus [74,75,76]. Hergenhahn and colleagues [77] have proved that curcumin (15 µM) inhibited the EBV reactivation in Raji DR-CAT cells by a mechanism of suppressing BZLF1 gene transcription. 

BoHV-1 is an enveloped DNA herpesvirus that infects cattle and causes a severe respiratory infection, leading to significant economic losses for the cattle industry [78,79]. In an in vitro experiment using Madin-Darby Bovine Kidney (MDBK) cells, curcumin (10 µM) substantially reduced viral titer, leading to inhibiting viral replication. The authors suggested that the anti-BoHV-1 activity of curcumin could be related to its capability to upregulate lipid raft formation [80]. On the other hand, co-encapsulation of acyclovir and curcumin into three microparticle formulations composed of the polymers hydroxypropyl methylcellulose, Eudragit^®^ RS100, or both were recently developed by Reolon et al. [81] for improving the antiviral efficacy against BoVH-1 infection. All three microparticle formulations were tested in infected MDBK cells with BoVH-1 and were observed to reduce the viral plaque formation at a concentration of 75 µg/mL. 

PRV is an infectious herpesvirus that belongs to the *Alphaherpesvirinae* subfamily. This virus is an etiological agent of Aujeszky’s disease, a viral infection that primarily affects pigs, causing reproductive and severe neurological complications in affected animals [82,83]. By employing an in vitro assay, the anti-PRV activity of curcumin was studied by infecting the porcine kidney (PK-15) cells with PRV and then treating them with curcumin at a concentration of 30 µM. The research demonstrated that curcumin effectively inhibited the infectivity of PRV by reducing viral plaque formation. On the other hand, the results did not report any mechanism of action [84]. 

## 7. Curcumin and Inflammatory Response to Herpesvirus Infections

**Figure 4:**
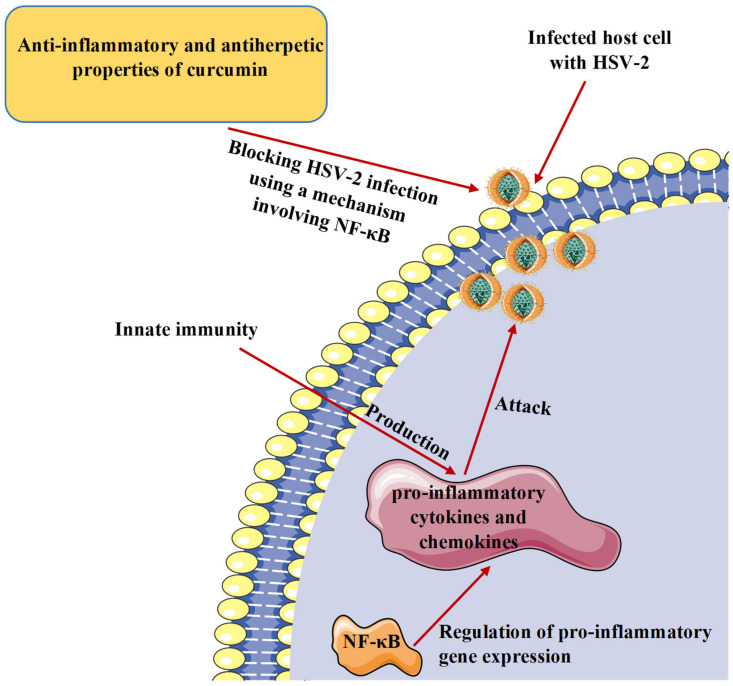
Protective effect of curcumin against herpes simplex virus and inflammation associated with this infection. HSV-2, herpes simplex virus 2; NF-κB, nuclear factor kappa B.

It is recognized that pro-inflammatory cytokines generated by activated innate immune cells are produced as a response to viral infection to stop the pathogen attack and restrict the replication process. However, pro-inflammatory cytokines have a negative effect as they play a vital role in inflammatory diseases of infectious origin [85,86]. The transcription factor nuclear factor kappa B (NF-κB) is known to regulate the expression of various pro-inflammatory genes, including those encoding cytokines and chemokines, and contributes to inflammasome regulation [87,88]. Curcumin has been shown to exhibit anti-inflammatory activities and regulate multiple cell signaling factors such as pro-inflammatory cytokines such as tumor necrosis factor-α (TNF-α), interleukin-1β (IL-1β), and interleukin-6 (IL-6) as well as transcription factors such as NF-κB and activator protein-1 (AP-1) [89,90]. In a study operated on HSV-2, curcumin has successfully blocked the virus infection through a mechanism involving NF-κB [52]. Considering the anti-inflammatory and antiherpetic properties, curcumin could be an effective drug to manage or prevent inflammation associated with herpesvirus infections (Figure 4).

## 8. Safety Profile and Reported Undesirable Effects in Clinical Studies

The utilization of curcumin has been confirmed with no toxicity, and the U.S. Food and Drug Administration (FDA) classified it as a ‘’Generally Recognized as Safe’’ (GRAS) compound [91,92]. Based on numerous clinical studies, curcumin has been shown to authenticate an outstanding safety profile and tolerability with no toxicity even at high oral doses of up to 12 g/day [93]. Moreover, the allowable daily intake value (0–3 mg/kg body weight) was recommended and authorized by the Joint Food and Agriculture Organization of the United Nations (FAO)/World Health Organization (WHO) Expert Committee on Food Additives (JECFA) and European Food Safety Authority (EFSA) [94]. Although curcumin has a marked safety record, some clinical observations have documented a few adverse effects on subjects treated with this molecule. For instance, diarrhea, headache, rash, and yellow stool were noted with seven subjects who received 1–12 g of curcumin in a dose-response study and monitored for 72 h [93]. In another clinical investigation, some negative effects such as nausea and diarrhea as well as an accretion in serum alkaline phosphatase and lactate dehydrogenase contents were detected with subjects received 0.45 to 3.6 g/day curcumin for 1–4 months [95].

## 9. Challenges with Bioavailability and Developed Strategies

**Figure 5:**
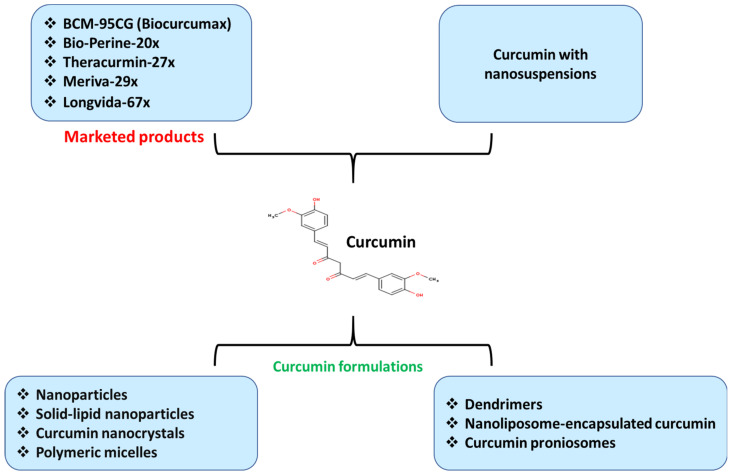
Developed drug delivery strategies for enhancing curcumin bioavailability using various types of curcumin formulations assisted by nanotechnology for the treatment of herpesvirus infections and their linked inflammation. Various marketed products with increased absorption and/or bioavailability of curcumin are presented.

The multipotent biological properties of curcumin against a wide range of diseases, including viral infections, have made this substance a promising drug for the treatment of herpesvirus diseases [96,97]. Nevertheless, curcumin has been reported to have weak bioavailability, which drives to low serum concentrations, diminishing the utilization of its potentially positive therapeutic effects [98]. The low bioavailability has been noted to be correlated with the insolubility in the water (pH = 7) and the potential degradation or crystallization in alkaline and acidic environments, respectively [99]. Over the past 20 years, numerous pharmaceutical approaches were successfully developed to enhance curcumin’s oral bioavailability by producing effective curcumin formulations such as BCM-95CG (Biocurcumax) Bio-Perine-20×, Theracurmin-27×, Meriva-29×, and Longvida-67× [100,101,102,103] (Figure 5). These products are currently accessible in markets with improved absorption and/or bioavailability of curcumin [103]. 

It is known that drug delivery is the process of administering a pharmaceutical substance to attain a therapeutic impact on humans or animals, using various approaches, formulations, and technologies, leading to overcoming many obstacles, including low bioavailability and weak absorption. Thus, several other efficient drug delivery systems that combine curcumin with nanosuspensions were generated [104]. Moreover, the recent advances in nanotechnology assisted in solving the challenges that face curcumin drug delivery by exploiting various nano-carriers such as nanoparticles and solid-lipid nanoparticles [105,106,107], curcumin nanocrystals [108], polymeric micelles [109], dendrimers, nanoliposome-encapsulated curcumin, and curcumin proniosomes [110,111]. We highly recommend that readers refer to the cited references to get more detailed information about the mentioned approaches, formulations, and technologies, which were employed to enhance the bioavailability and absorption of curcumin along with the used mechanisms.

## 10. New Directions for Herpesviruses Treatment

Recently, the CRISPR/Cas9 genome editing technique has been developed as a new strategy that targets viral genetic elements essential for viral fitness. This technique has been developed so that it does not require active replication to perform, and therefore, it is valuable to tackle both productive and latent herpesvirus infections, leading to eradicating viral production from infected cells [112]. This approach has been employed in the genome editing and blocking of latent infections for herpesviruses such as HSV, EBV, KSHV, and HCMV [113,114]. On the other hand, CRISPR/Cas9-mediated genome editing system combined with antiherpetic drugs (natural or synthetic) may significantly change the future of the treatment of herpesviruses and overcome the complications connected with their post-infections.

## 11. Conclusion and Future Visions

Combating viral diseases, especially those triggered by herpesviruses, has always been a challenge, and the long-term administration of antiherpetic drugs has led to generating various undesirable effects and the problem of drug resistance. Therefore, there is an imperative need for new drug candidates with functional properties to conquer these complications. Plants and their active ingredients are ideal screening libraries for antiviral agents because of their advantages, such as convenient acquisition and low side effects. In recent years, curcumin as a nutraceutical agent has attracted major attention in many research fields due to its great therapeutic potential against various biological targets. Hence, in this paper, we have comprehensively reviewed the curative values of curcumin against numerous animal and human herpesviruses along with the mechanisms by which this compound induces antiherpetic properties, which were examined in vitro and in vivo investigations. Based on several structure-activity relationship studies, hydroxyl groups, carbonyl groups, and phenyl rings of curcumin were observed to be accountable for the induced anti-herpesvirus properties. 

Moreover, this paper highlighted several developed strategies to improve curcumin’s bioavailability and antiviral properties against various types of herpesvirus. These approaches made this drug a promising candidate to be involved in clinical studies with enhanced anti-herpesvirus activities. On the other hand, the possible combinatory treatment of curcumin with various antiherpetic nucleoside analogs might be another useful option for the therapy of herpesvirus infections; however, these investigations are still limited or have yet to be proven in vivo. 

In conclusion, this review proposes curcumin as a potent and safe drug for the therapy of herpesvirus infections as well as inflammation associated with these infections.

## Figures and Tables

**Table 1:** Antiviral activities of curcumin, curcumin metal complexes, and curcumin formulations against herpes simplex virus 1 (HSV-1) and herpes simplex virus 2 (HSV-2) infections.

Type of Study, Test Performed, Virus, and Cells/Animal Model	Results	Mechanism of Action and Pathway	Reference
In vitro. Plaque assays, ChIP assay, and western blot analysis. HSV-1. HeLa and Vero cells.	Inhibition of HSV-1 replication and suppression of IE gene expression.	Curcumin was observed to utilize the mechanism independent of the transcriptional coactivator proteins p300/CBP histone acetyltransferase activity to affect the viral transactivator protein VP16-mediated enlistment of RNA polymerase II to IE gene promoters, leading to suppressing gene expression and blocking viral infection.	[50]
In vitro and in vivo. Plaque reduction assay. HSV-2. Primary rabbit kidney cells (in vitro). Guinea pig model (in vivo).	In an in vitro plaque reduction assay, curcumin suppressed the replication of HSV-2 with an ED_50_ value of 0.32 mg/mL, while at a concentration of 100 mg/mL, the in vivo inhibitory activity was confirmed using a mouse model of genital HSV-2 infection.	The mechanism is unknown.	[51]
In vitro. Plaque assay. HSV-2. Human genital epithelial cells.	In primary human genital epithelial cells, pre-treatment of cells with curcumin (5 µM) decreased HSV-2 shedding by 1000-fold and at a concentration of 50 µM, entirely blocked HSV-2 production.	Investigation of the cellular pathways known to be regulated by curcumin involving the transcription factor NF-κB.	[52]
In vitro. Plaque assay and virus adsorption assay. HSV-1 and HSV-2. Vero cells.	At a concentration of 30 µM, curcumin inhibited the replication of HSV-1 and HSV-2.	Inhibition of adsorption and replication of HSV-1 and HSV-2.	[53]
In vivo. Plaque assay. HSV-2. Genital epithelial cells of female C57BL/6 mice.	Nanoparticle-containing curcumin (0.5 mg) reduced tissue inflammation and the severity of HSV-2 infection in an animal model.	The mechanism of action was detected to be correlated with the anti-inflammatory properties of curcumin.	[54]
In vitro. Cytopathic inhibition assay. HSV-1. Vero cells.	Curcumin, gallium-curcumin, and copper-curcumin inhibited the replication of HSV-1 with IC_50_ values of 33.0, 13.9, and 23.1 µg/mL, respectively.	The mechanisms of action of both gallium-curcumin and copper-curcumin have been suggested to be investigated in further studies.	[55]

CBP, CREB-binding protein; ChIP, chromatin immunoprecipitation; ED_50_, the concentration of drug that decreased the plaque number by 50%; HSV-1, herpes simplex virus 1; HSV-2, herpes simplex virus 2; IC_50_, 50% inhibitory concentration; IE gene, immediate early gene; NF-κB, nuclear factor kappa B.

**Table 2:** Antiviral properties of curcumin and curcumin formulations against human cytomegalovirus (HCMV), Kaposi’s sarcoma-associated herpesvirus (KSHV), Epstein–Barr virus (EBV), bovine herpesvirus 1 (BoHV-1), and pseudorabies virus (PRV) infections.

Herpesvirus and Type of Study	Results	Mechanism of Action and Pathway	Reference
HCMV (in vitro, in vivo, and in silico).	At various concentrations in micromolar ranges, curcumin was detected with anti-HCMV properties.	Inhibition of IEA and UL83A expressions and downregulation of Hsp90. Determination of anti-inflammatory and antioxidant effects as possible mechanisms underlying the anti-HCMV activity.	[66,67,68]
KSHV (in vitro).	At various concentrations (in µM), curcumin efficiently inhibited KSHV replication and virus-associated pathogenic properties.	Blocking APE1-mediated redox function.	[73]
EBV (in vitro).	Inhibition of EBV reactivation in Raji DR-CAT cells with curcumin treatment (15 µM).	Inhibition of BZLF1 gene transcription.	[77]
BoHV-1 (in vitro).	At a concentration of 10 µM, curcumin reduced BoHV-1 titer, leading to inhibiting viral replication. Co-encapsulation of acyclovir and curcumin into three microparticle formulations noticeably reduced the BoVH-1 plaque formation at a concentration of 75 µg/mL.	Inhibition of virus post-binding entry process by upregulating the lipid raft formation.	[80,81]
PRV (in vitro).	Treatment with curcumin (30 µM) blocked PRV infectivity in PK-15 cells by decreasing the viral plaque formation.	No mechanism of action was revealed.	[84]

APE1, apurinic/apyrimidinic endonuclease 1; BoHV-1, bovine herpesvirus 1; EBV, Epstein–Barr virus; HCMV, human cytomegalovirus; Hsp90, heat shock protein 90; IEA, immediate early antigen; KSHV, Kaposi’s sarcoma-associated herpesvirus; PK, porcine kidney; PRV, pseudorabies virus.

## Data Availability

Not applicable.
